# Sex differences in heart rate and heart rate variability in rats: Implications for translational research

**DOI:** 10.3389/fphys.2023.1170320

**Published:** 2023-03-24

**Authors:** Luca Carnevali, Margherita Barbetti, Rosario Statello, DeWayne P. Williams, Julian F. Thayer, Andrea Sgoifo

**Affiliations:** ^1^ Stress Physiology Lab, Department of Chemistry, Life Sciences and Environmental Sustainability, University of Parma, Parma, Italy; ^2^ Department of Medicine and Surgery, University of Parma, Parma, Italy; ^3^ Department of Medical Sciences and Public Health, University of Cagliari, Cagliari, Italy; ^4^ Department of Psychological Science, University of California, Irvine, Irvine, United States

**Keywords:** vagal, rats, sex differences, heart rate, heart rate variabiity (HRV)

## Abstract

The present study aimed to investigate sex differences in measures of cardiac chronotropy and heart rate variability (HRV) in 132 young adult wild-type Groningen rats (*n* = 45 females). Electrocardiographic signals were recorded for 48 h in freely moving rats to quantify heart rate (HR) and inter-beat interval (IBI) as measures of cardiac chronotropy, and time- and frequency-domain HRV parameters as physiological readouts of cardiac vagal modulation. Females showed greater vagally-mediated HRV despite having higher HR and shorter IBI than males during undisturbed conditions. Such differences were evident i) at any given level of HRV, and ii) both during the 12-h light/inactive and 12-h dark/active phase of the daily cycle. These findings replicate the paradoxical cardiac chronotropic control reported by human meta-analytic findings, since one would expect greater vagally-mediated HRV to be associated with lower HR and longer IBI. Lastly, the association between some HRV measures and HR was stronger in female than male rats. Overall, the current study in young adult rats provides data illustrating a sex-dependent association between vagally-mediated HRV and indexes of cardiac chronotropy. The current results i) are in line with human findings, ii) suggest to always consider biological sex in the analysis and interpretation of HRV data in rats, and iii) warrant the use of rats for investigating the neuro-hormonal basis and temporal evolution of the impact of sex on the association between vagally-mediated HRV and cardiac chronotropy, which could inform the human condition.

## 1 Introduction

It is widely known that parasympathetic (vagal) modulation exerts a negative chronotropic effect on the heart by slowing spontaneous depolarization of the pacemaker cells in the sinoatrial node (Bartos et al., 2015). Cardiac vagal modulation can be indirectly assessed through analysis of heart rate variability (HRV), as shown by pharmacological studies suggesting that vagal influences to sinoatrial node activity are responsible for HRV within the respiratory frequency band (McCabe et al., 1985; Pagani et al., 1986; Japundzic et al., 1990). Therefore, the association of respiratory-linked HRV with vagal influence as its putative mechanism has led to the use of HRV metrics as an approximation of cardiac vagal modulation (Laborde et al., 2017). Thus, one would expect greater vagally-mediated (vm) HRV to be associated with lower heart rate (HR) and longer inter-beat interval (IBI). However, in humans there is an apparent sex paradox in the relationship between resting measures of vmHRV and indexes of cardiac chronotropy. Specifically, meta-analytic findings showed women to have greater vmHRV and, paradoxically, higher HR and lower IBI compared to men (Koenig and Thayer, 2016). Further, a recent study conducted on a large sample (*n* = 628) of young adults demonstrated stronger associations between resting measures of vmHRV and both HR and IBI in women than man (Williams et al., 2022). According to the authors, this finding suggests that vagal modulation (as indexed by vmHRV) is of greater impact on cardiac chronotropic control (i.e., HR and IBI) in women compared to men, which may be due to the effects of sex hormones, such as estradiol, on the sensitivity to the cholinergic neurotransmitter acetylcholine (ACh) (Du et al., 1994; Dart et al., 2002). Thus, given the popularity and feasibility of HRV research nowadays, they recommended considering gender as a fundamental covariate in HRV research (Koenig and Thayer, 2016; Williams et al., 2022).

The use of HRV as a non-invasive physiological read-out of cardiac vagal modulation has become popular also in rodent research to increase the knowledge of several (patho)physiological processes (e.g., Wood et al., 2012; Lee et al., 2013; Carnevali and Sgoifo, 2014; Chuang et al., 2017; Morais-Silva et al., 2019). Yet, much uncertainty remains in the translational relevance of the results. One of the most important criticisms concerns the fact that sympatho-vagal contributions to cardiac chronotropy may vary significantly between humans and rodents. For example, in humans resting HR is largely determined by vagal modulation (Tan et al., 2009), while in mice and rats vagal contributions to resting HR seem less predominant (Japundzic et al., 1990; Ishii et al., 1996; Gehrmann et al., 2000; Carnevali and Sgoifo, 2014; Axsom et al., 2020). Therefore, one may argue that rodent-based findings on resting measures of vmHRV have little translational relevance for the human condition. Nevertheless, several studies have reported that in rodents, like in humans, resting measures of vmHRV i) decline with advancing age (Rossi et al., 2014; Piantoni et al., 2021), ii) may be important predictors of ventricular arrhythmic risk (Carnevali et al., 2019), iii) are influenced by environmental factors such as stress exposure (Grippo et al., 2002; Wood et al., 2012), and iv) are associated with specific behavioral phenotypes (e.g., Grippo et al., 2002; Wood et al., 2012; Carnevali et al., 2013a; Carnevali et al., 2014). Importantly, most of these studies have been conducted on male rodents, and none has investigated the potential impact of biological sex on vmHRV and its association with indexes of cardiac chronotropy in freely moving rodents. Such an investigation would be useful for providing further support to the translational value of HRV findings in rodent models and for gaining new knowledge on sex differences in cardiac chronotropic control. Therefore, in the present study we assessed measures of HRV and cardiac chronotropy from 24-h radiotelemetric ECG recordings in young adult male and female rats. Based on human findings (Koenig and Thayer, 2016; Williams et al., 2022), we hypothesized that i) female rats would show greater vmHRV and, paradoxically, higher HR - and lower IBI - than male rats, and that ii) the association between vmHRV and HR/IBI would be stronger in female than male rats.

## 2 Materials and methods

### 2.1 Animals

Three-month-old wild-type Groningen rats were considered for the present investigation. This rat strain, originally derived from the University of Groningen (Netherlands), is currently bred at the University of Parma in climate-controlled rooms, with a 12-h light/dark cycle (lights on at 7 p.m.) and *ad libitum* food and water. Data were pooled across seven studies conducted within our lab (Carnevali et al., 2012; Carnevali et al., 2013a; Carnevali et al., 2013b; Carnevali and Sgoifo, 2014; Carnevali et al., 2020; Andolina et al., 2021; and one ongoing study). There were 87 males and 45 females available for the analysis. Each study was approved by the Italian legislation on animal experimentation (D.L. 04/04/2014, n. 26, authorization of the ongoing study n.473/2022-PR).

### 2.2 ECG recordings and analysis

In each experiment, rats were implanted under anesthesia with radiotelemetric transmitters (TA11CTA-F40, Data Sciences International, St. Paul, MN) for recordings of ECG signals (sampling frequency 1,000 Hz). The transmitter body was placed in the abdominal cavity; one electrode was fixed to the dorsal surface of the xyphoid process and another electrode was placed in the anterior mediastinum close to the right atrium, according to the previously described procedure (Sgoifo et al., 1996). Animals were allowed a 2-week recovery period before the beginning of ECG recordings.

For the analysis of ECG signals, we considered 2-min segments recorded every hour for two consecutive days during undisturbed conditions and before the execution of any other experimental procedure (e.g., stress protocols) adopted in the original studies. Initially, each raw ECG segment was visually inspected to ensure that all R-waves were correctly detected. Those parts of ECG traces which exhibited recording artifacts or arrhythmias were discarded without substitution and excluded from further analysis. ECG segments were then analyzed for the present investigation using ChartPro 5.0 software (ADInstruments, Sydney, Australia). For each 2-min segment, mean HR (reported in beats per minute, bpm), IBI (ms), and time- and frequency-domain parameters of HRV were quantified. In the time-domain, we considered the standard deviation of IBIs (SDNN, ms), which reflects both vagal and sympathetic influences, and the root mean square of successive beat-to-beat interval differences (RMSSD, ms), which reflects vagal regulation of HR. For spectral (frequency-domain) analysis of HRV, a power spectrum was obtained with a fast Fourier transform-based method (Welch’s periodogram: 256 points, 50% overlap, and Hamming window). We considered the power (ms^2^) of the low frequency band (LF, 0.2–0.75 Hz), which reflects both vagal and sympathetic influences, and the power (ms^2^) of the high frequency band (HF, 0.75–2.5 Hz), which reflects vagal regulation of HR. In sum, measures of cardiac chronotropy included HR and IBI and measures of HRV included RMSSD and HF as indexes of vagal modulation and SDNN and LF as indexes of both sympathetic and vagal influences. HR, IBI, and HRV data were averaged to obtain 24-h values and 12-h values for the light and dark phases of the daily cycle.

### 2.3 Statistical analysis

All statistical analyses were performed using the IBM SPSS statistical package (version 28). The normal distribution of variables was checked by means of the Kolmogorov–Smirnov test. Significantly skewed variables, including SDNN, RMSSD, LF and HF were log transformed (ln) to fit assumptions for linear analyses.

A series of Student’s t-tests for independent samples were applied to test potential differences between male and female rats on all variables. Zero-order correlations (Pearson’s r) were computed to investigate the relationship between HRV parameters and measures of cardiac chronotropy in the full sample and, separately, in the two sexes. Sex differences in correlation coefficients were tested using Fisher’s r-to-z transformation (Steiger, 1980).

Then, based on (Williams et al., 2022), the SPSS macro “PROCESS” was used to test if sex moderated the relationship between HRV indexes and cardiac chronotropy. Specifically, “Model 1” was used to test the interactive effect of 24-h HRV measures (independent variable) and sex (moderator, 1 = males, 2 = females) on 24-h HR values. Conditional effects were used to determine the differential relationship between male and female rats on the association between 24-h HRV parameters and 24-h HR using simple slope analyses. The Johnson-Neyman technique was applied to identify regions of significance and determine how male and female rats differed in HR at low, mean, and high levels of HRV (predictor), with high and low HRV values that were derived using ±1SD from the mean. Statistical significance was set at *p* < 0.05.

## 3 Results

### 3.1 Sex differences in indexes of cardiac chronotropy and HRV

**FIGURE 1:**
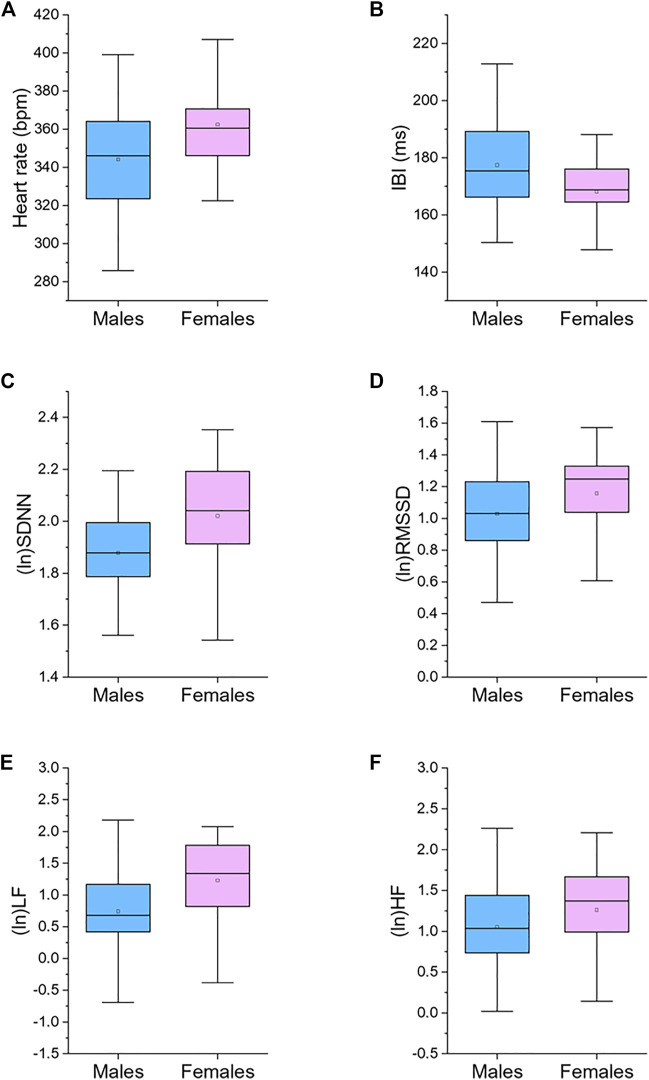
Box plots for 24-h values of heart rate **(A)**, inter-beat interval (IBI, **(B)**), standard deviation of IBIs (SDNN, **(C)**), root mean square of successive beat-to-beat interval differences (RMSSD, **(D)**), low frequency power (LF, 0.2–0.75 Hz; **(E)**), and high frequency power (HF, 0.75–2.5 Hz; **(F)**), divided by male (*n* = 87) and female (*n* = 45) rats. The boxes show the data between the 25th and 75th percentile, the middle line represents the median, the black dot represents the mean. Ln, natural logarithm. Statistics are reported in Table 1.

Table 1 reports 24-h values of cardiac chronotropy and HRV and separate estimates for the 12-h light and 12-h dark phase of the daily cycle in male and female rats. Box and whisker plots in Figure 1 show representative distributions of 24-h measures of cardiac chronotropy and HRV in both sexes.

**TABLE 1:** Mean differences between male and female rats on 24-h values and on 12-h values for the light (inactive) and dark (active) phases of the daily cycle.

	Males (n = 87)	Females (n = 45)	t	p	d
HR (bpm) 24-h	344.1 ± 27.1	362.5 ± 21.9	−3.93	<0.001	−0.72
HR (bpm) 12-h light	326.4 ± 29.0	346.6 ± 22.9	−4.01	<0.001	−0.74
HR (bpm) 12-h dark	361.5 ± 29.8	378.5 ± 23.6	−3.32	<0.001	−0.61
IBI (ms) 24h	177.4 ± 15.0	168.1 ± 10.1	4.23	<0.001	0.69
IBI (ms) 12-h light	186.1 ± 18.1	175.6 ± 11.3	4.07	<0.001	0.65
IBI (ms) 12-h dark	168.9 ± 15.3	160.7 ± 10.3	3.64	<0.001	0.59
SDNN (ms) 24-h	6.65 ± 1.17	7.71 ± 1.56	−3.89	<0.001	−0.78
SDNN (ms) 12-h light	6.93 ± 1.33	8.02 ± 1.68	−3.57	<0.001	−0.73
SDNN (ms) 12-h dark	6.37 ± 1.31	7.40 ± 1.58	−3.62	<0.001	−0.72
(ln)SDNN 24-h	1.88 ± 0.19	2.02 ± 0.22	−3.53	<0.001	−0.71
(ln)SDNN 12-h light	1.92 ± 0.20	2.06 ± 0.22	−3.40	<0.001	−0.68
(ln)SDNN 12-h dark	1.83 ± 0.21	1.98 ± 0.24	−3.29	<0.001	−0.66
RMSSD (ms) 24-h	2.89 ± 0.71	3.30 ± 0.82	−3.04	0.003	−0.56
RMSSD (ms) 12-h light	3.06 ± 0.83	3.45 ± 0.92	−2.47	0.015	−0.45
RMSSD (ms) 12-h dark	2.71 ± 0.68	3.15 ± 0.84	−3.24	0.002	−0.59
(ln)RMSSD 24-h	1.03 ± 0.25	1.16 ± 0.30	−2.59	0.011	−0.48
(ln)RMSSD 12-h light	1.08 ± 0.27	1.20 ± 0.31	−2.18	0.031	−0.40
(ln)RMSSD 12-h dark	0.97 ± 0.26	1.11 ± 0.31	−2.76	0.007	−0.51
LF (ms^2^) 24-h	2.45 ± 1.42	4.13 ± 2.12	−4.79	<0.001	−1.00
LF (ms^2^) 12-h light	2.74 ± 1.69	4.34 ± 2.40	−3.97	<0.001	−0.81
LF (ms^2^) 12-h dark	2.15 ± 1.35	3.92 ± 2.23	−4.87	<0.001	−1.04
(ln)LF 24-h	0.74 ± 0.56	1.23 ± 0.73	−4.24	<0.001	−0.78
(ln)LF 12-h light	0.83 ± 0.62	1.26 ± 0.74	−3.50	<0.001	−0.64
(ln)LF 12-h dark	0.60 ± 0.59	1.14 ± 0.78	−4.52	<0.001	−0.83
HF (ms^2^) 24-h	3.23 ± 1.66	4.07 ± 1.94	−2.58	0.011	−0.47
HF (ms^2^) 12-h light	3.74 ± 2.20	4.59 ± 2.49	−2.01	0.046	−0.37
HF (ms^2^) 12-h dark	2.73 ± 1.42	3.54 ± 1.76	−2.88	0.005	−0.53
(ln)HF 24-h	1.05 ± 0.51	1.26 ± 0.61	−2.11	0.037	−0.39
(ln)HF 12-h light	1.17 ± 0.54	1.36 ± 0.64	−1.74	0.085	−0.32
(ln)HF 12-h dark	0.87 ± 0.52	1.11 ± 0.64	−2.15	0.024	−0.42

Note. Data are reported as mean ± SD., Independent samples *t*-test include both t- and *p*-values in addition to Cohen’s d. Abbreviations: HR, heart rate; IBI, inter-beat interval; SDNN, standard deviation of IBIs; RMSSD, root mean square of successive beat-to-beat interval differences; LF, low frequency power (0.2–0.75 Hz); HF, high frequency power (0.75–2.5 Hz); ln, natural logarithm.

Female rats showed higher 24-h HR and lower 24-h IBI compared to male rats (Table 1; Figure 1). These significant differences emerged both during the light and dark phase of the daily cycle (Table 1). Female rats had also higher 24-h values of SDNN, natural log-transformed SDNN, RMSSD, natural log-transformed RMSSD, LF power, natural log-transformed LF, HF power, and natural log-transformed HF (Table 1; Figure 1). These sex differences in HRV values were significant during both the 12-h light and 12-h dark phase of the daily cycle, with the only exception of (ln)HF light values (*p* = 0.085) (Table 1).

### 3.2 Correlations between indexes of cardiac chronotropy and HRV

Table 2 reports correlation coefficients for 24-h values of cardiac chronotropy and HRV in the full sample and stratified by sex. In the full sample, there was a near-perfect negative association between HR and IBI (r = 0.993) and a near-perfect positive association between the two indexes of vagally-mediated HRV (natural log-transformed RMSSD and HF, r = 0.983) (Table 2 A). As expected, higher HR and lower IBI were significantly associated with lower vagally-mediated HRV (natural log-transformed RMSSD and HF values) (Table 2 A). Significant and strong positive correlations were also found between time-domain indexes of HRV (natural log-transformed SDNN and RMSSD, r = 0.690), and between frequency-domain indexes of HRV (natural log-transformed LF and HF, r = 0.824). This result confirms that, besides the two well-accepted indexes of vagally-mediated HRV (i.e., RMSSD and HF), cardiac vagal influences in the rat are also largely captured by SDNN and LF values. As for sex differences, female rats tended to have a stronger negative correlation between (ln)RMSSD and HR than males (r = −0.534 vs. r = −0.394), although this difference did not reach statistical significance (*p* > 0.05) (Table 2 A, B). Notably, a significant negative correlation was found between (ln)SDNN and HR in females (r = −0.544, *p* < 0.01) (Table 2 C), but not in male rats (r = −0.172, *p* > 0.05) (Table 2 B), with correlation coefficients that differed significantly between sexes (*p* = 0.02). Similarly, a significant negative correlation was found between (ln)LF and HR in females (r = −0.451, *p* < 0.01) (Table 2 C), but not in male rats (r = −0.207, *p* > 0.05) (Table 2 B), although sex differences in correlation coefficients did not reach statistical significance (*p* = 0.14). The same results were obtained when IBI, rather than HR, was used as index of cardiac chronotropy. Further, results were consistent when correlation coefficients were computed separately for the 12-h light (Table 3) and 12-h dark (Table 4) phases of the daily cycle.

**TABLE 2:** Correlation coefficients in the full sample and stratified by sex for 24-h values of cardiac chronotropy and heart rate variability.

A: Full sample	1	2	3	4	5	6
1. 24-h HR	-					
2. 24-h IBI	−.993**	-				
3. 24-h (ln)SDNN	−.118	.103	-			
4. 24-h (ln)RMSSD	−.327**	.330**	.690**	-		
5. 24-h (ln)LF	−.138	.133	.870**	.831**	-	
6. 24-h (ln)HF	−.378**	.381**	.692**	.983**	.824**	-
B: Males						
1. 24-h HR	-					
2. 24-h IBI	−.995**	-				
3. 24-h (ln)SDNN	−.172	.186	-			
4. 24-h (ln)RMSSD	−.394**	.414**	.552**	-		
5. 24-h (ln)LF	−.207	.212*	.822**	.771**	-	
6. 24-h (ln)HF	−.462**	.478**	.561**	.978**	.762**	-
C: Females						
1. 24-h HR	-					
2. 24-h IBI	−.996**	-				
3. 24-h (ln)SDNN	−.544**	.553**	-			
4. 24-h (ln)RMSSD	−.534**	.519**	.811**	-		
5. 24-h (ln)LF	−.451**	.445**	.880**	.895**	-	
6. 24-h (ln)HF	−.512**	.501**	.832**	.990**	.907**	-

Note. in the table, A represents the correlation coefficients between the variables of interest for the full sample (*n* = 132), B and C represent these correlation coefficients split by male (n = 87) and female (n = 45) rats. Abbreviations: HR, heart rate; IBI, inter-beat interval; SDNN, standard deviation of IBIs; RMSSD, root mean square of successive beat-to-beat interval differences; LF, low frequency power (0.2–0.75 Hz); HF, high frequency power (0.75–2.5 Hz); ln, natural logarithm. *p < 0.05 and **p < 0.01.

**TABLE 3:** Correlation coefficients in the full sample and stratified by sex for 12-h values of cardiac chronotropy and heart rate variability during the light phase of the daily cycle.

A: Full sample	1	2	3	4	5	6
1. 12-h HR	-					
2. 12-h IBI	−.962**	-				
3. 12-h (ln)SDNN	−.127	.166	-			
4. 12-h (ln)RMSSD	−.303**	.333**	.734**	-		
5. 12-h (ln)LF	−.120	.139	.876**	.815**	-	
6. 12-h (ln)HF	−.359**	.379**	.736**	.982**	.814**	-
B: Males						
1. 12-h HR	-					
2. 12-h IBI	−.955**	-				
3. 12-h (ln)SDNN	−.251	.326*	-			
4. 12-h (ln)RMSSD	−.382**	.431**	.659**	-		
5. 12-h (ln)LF	−.200	.230*	.845**	.762**	-	
6. 12-h (ln)HF	−.454**	.487**	.670**	.977**	.767**	-
C: Females						
1. 12-h HR	-					
2. 12-h IBI	−.995**	-				
3. 12-h (ln)SDNN	−.449**	.459**	-			
4. 12-h (ln)RMSSD	−.441**	.426**	.821**	-		
5. 12-h (ln)LF	−.340*	.331*	.880**	.876**	-	
6. 12-h (ln)HF	−.430**	.417**	.838**	.991**	.878**	-

Note. in the table, A represents the correlation coefficients between the variables of interest for the full sample (*n* = 132), B and C represent these correlation coefficients split by male (*n* = 87) and female (*n* = 45) rats. Abbreviations: HR, heart rate; IBI, inter-beat interval; SDNN, standard deviation of IBI; RMSSD, root mean square of successive beat-to-beat interval differences; LF, low frequency power (0.2–0.75 Hz); HF, high frequency power (0.75–2.5 Hz); ln, natural logarithm. *p < 0.05 and **p < 0.01.

**TABLE 4:** Correlation coefficients in the full sample and stratified by sex for 12-h values of cardiac chronotropy and heart rate variability during the dark phase of the daily cycle.

A: Full sample	1	2	3	4	5	6
1. 12-h HR	-					
2. 12-h IBI	−.971**	-				
3. 12-h (ln)SDNN	−.134	.162	-			
4. 12-h (ln)RMSSD	−.329**	.322**	.646**	-		
5. 12-h (ln)LF	−.164	−.140	.826**	.813**	-	
6. 12-h (ln)HF	−.371**	.346**	.628**	.978**	.817**	-
B: Males						
1. 12-h HR	-					
2. 12-h IBI	−.965**	-				
3. 12-h (ln)SDNN	−.136	.237	-			
4. 12-h (ln)RMSSD	−.357**	.365**	.505**	-		
5. 12-h (ln)LF	−.215*	.191	.729**	.774**	-	
6. 12-h (ln)HF	−.416**	.393**	.467**	.971**	.780**	-
C: Females						
1. 12-h HR	-					
2. 12-h IBI	−.995**	-				
3. 12-h (ln)SDNN	−.563**	.566**	-			
4. 12-h (ln)RMSSD	−.581**	.576**	.764**	-		
5. 12-h (ln)LF	−.482**	.481**	.880**	.844**	-	
6. 12-h (ln)HF	−.555**	.552**	.781**	.987**	.864**	-

Note. in the table, A represents the correlation coefficients between the variables of interest for the full sample (*n* = 132), B and C represent these correlation coefficients split by male (*n* = 87) and female (*n* = 45) rats. Abbreviations: HR, heart rate; IBI, inter-beat interval; SDNN, standard deviation of IBIs; RMSSD, root mean square of successive beat-to-beat interval differences; LF , low frequency power (0.2–0.75 Hz); HF, high frequency power (0.75–2.5 Hz); ln, natural logarithm. *p < 0.05 and **p < 0.01.

### 3.3 Prediction of HR by HRV indexes and the moderating role of sex

**FIGURE 2:**
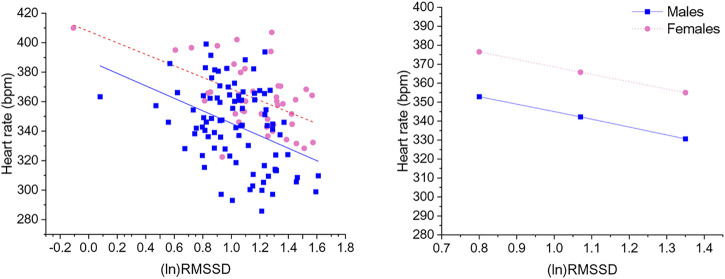
The left panel shows a scatterplot of 24-h values of (ln)RMSSD and heart rate as a function of sex. The right panel illustrates the prediction of heart rate values in both sexes at low, mean, and high (ln)RMSSD values. Higher and lower estimates of (ln)RMSSD were derived from ±1SD from the mean. (ln)RMSSD = natural log-transformed root mean square of successive beat-to-beat interval differences.

**FIGURE 3:**
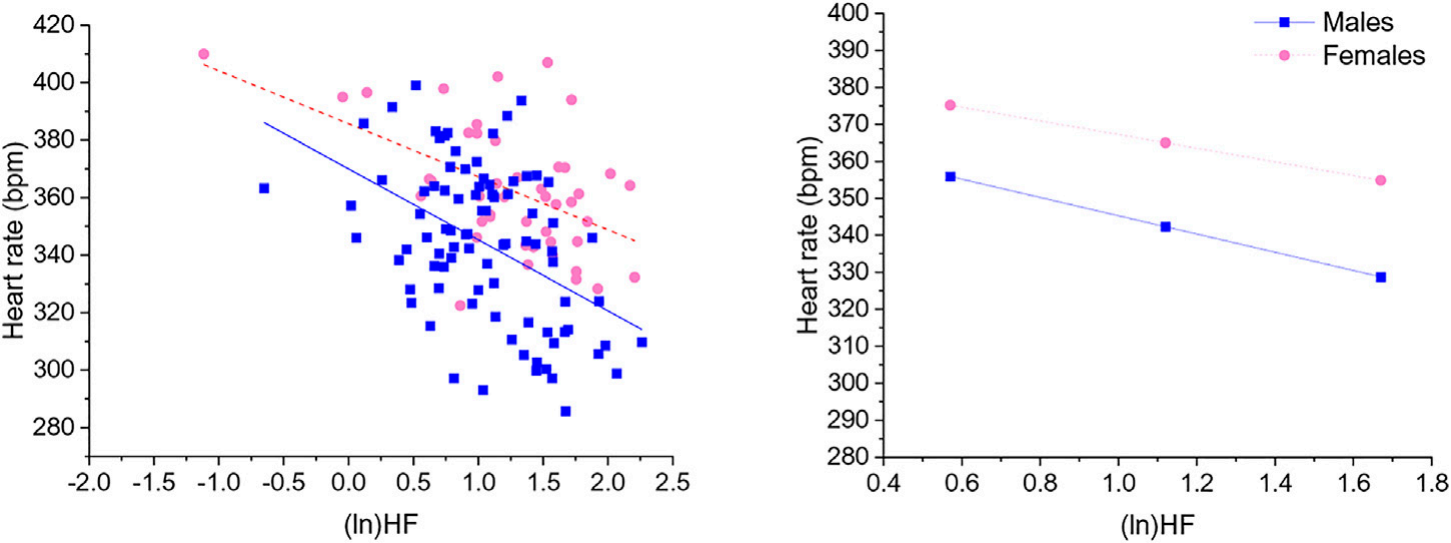
The left panel shows a scatterplot of 24-h values of (ln)HF and heart rate as a function of sex. The right panel illustrates the prediction of heart rate values in both sexes at low, mean, and high (ln)HF values. Higher and lower estimates of (ln)HF were derived from ±1SD from the mean. (ln)HF, natural log-transformed high frequency power.

Significant negative associations were found between (ln)RMSSD and HR [B = −45.5 (22.9) (confidence intervals: −90.9, −0.1], *p* < 0.05] and (ln)HF and HR [B = −30.9 (11.1) (confidence intervals: −52.9, −8.9), *p* < 0.05], independently from sex. The Johnson-Neyman technique revealed that in rats with (ln)RMSSD above 0.45 and (ln)HF above 0.17, respectively, females showed higher HR than males (Figures 2, 3). Sex significantly moderated the association between 24-h values of (ln)SDNN and HR [R^2^_Δ_ = 0.05, B = −30.1 (12.8) (confidence intervals: −75.4, −15.2), *p* < 0.05], such that female rats showed a stronger negative association [B = −54.2 (15.7) (confidence intervals: −85.5, −22.9), *p* < 0.05] compared to male rats [B = −24.1 (16.5) (confidence intervals: −56.8, 8.6), *p* > 0.05]. The Johnson-Neyman technique showed that in rats with (ln)SDNN below 2.33, females had higher HR than males. No significant associations were found between (ln)LF and HR.

## 4 Discussion

In this study, we analyzed 24-h ECG recordings in freely moving young adult wild-type Groningen rats to investigate the extent to which cardiac chronotropic control differs as a function of biological sex.

Our first hypothesis that female rats would show greater vmHRV (i.e., RMSSD and HF indexes) and higher HR—and lower IBI - compared to male rats was confirmed by the present results. These findings replicate the paradoxical cardiac chronotropic control observed in humans (Koenig and Thayer, 2016), since higher vmHRV is typically associated with lower HR and longer IBI. Notably, female rats had approximately 0.7 standard deviation (Cohen’s *d*) higher HR and lower IBI than males, with the magnitude of this sex difference being greater than those reported in a previous human meta-analysis (*Hedge’s g* = 0.36 for IBI) (Koenig and Thayer, 2016) and in a recent study in young adult humans (*d* = 0.30 for HR and *d* = 0.27 for IBI) (Williams et al., 2022). Further, female rats showed greater cardiac vagal modulation indexed by both RMSSD and HF power of HRV, while women were found to exhibit higher HF, but not RMSSD, values (Koenig and Thayer, 2016; Williams et al., 2022). Notably, while HR was higher in women compared to men especially at lower levels of vmHRV (Williams et al., 2022), here we show that at any given level of vmHRV (RMSSD and HF) female rats showed higher HR than male rats. Therefore, the sex paradox in cardiac chronotropic control (i.e., greater vmHRV but faster HR in females) seems more evident in this sample (*n* = 132) of young adult rats than that previously described in a large sample of young adult humans (*n* = 628, mean age = 19.22 ± 1.68 years) (Williams et al., 2022) and in the human meta-analysis (Koenig and Thayer, 2016). This could be due to the numerous anthropometric (e.g., body mass index), lifestyle (e.g., smoking; physical activity), and/or environmental (e.g., social stress) variables known to influence HRV in healthy subjects (Fagard et al., 1999; Aeschbacher et al., 2016; Kim et al., 2018), which could attenuate sex differences in humans but can clearly be controlled for and standardized in rats born and raised in a laboratory environment.

Of note, in a previous study we demonstrated that pharmacological blockade of cardiac vagal influences with the muscarinic receptor antagonist methylscopolamine provoked a nearly complete reduction of RMSSD values and HF power in the same wild-type Groningen strain used in the current investigation (Carnevali et al., 2013a). This strongly supports the use of RMSSD values and HF power of HRV as indexes of vagal modulation in rats, as in humans (Laborde et al., 2017). Relatedly, a strong positive correlation between vagus nerve electrical activity and HF power has been demonstrated in anesthetized rats (Kuo et al., 2005), although a recent study failed to replicate this finding but in a very small number of rats (Marmerstein et al., 2021). On the other hand, SDNN and LF power are generally thought to reflect both vagal and sympathetic influences in humans (Laborde et al., 2017), with contrasting views on whether LF power can be used as an index of baroreflex function (e.g., Rahman et al., 2011; Martelli et al., 2014). Nevertheless, men showed higher SDNN and LF power of HRV than women (Koenig and Thayer, 2016; Williams et al., 2022), whereas in this study in rats we observed the opposite phenomenon. To explain this discrepancy, we found strong correlations between time-domain HRV indexes (i.e., SDNN and RMSSD) and between frequency-domain HRV indexes (LF and HF power), suggesting that SDNN values and LF power mostly capture cardiac vagal modulation in rats. Relatedly, in a previous study in young adult mice, methylscopolamine administration provoked a drastic reduction not only in RMSSD values and HF power, but also in SDNN values and HF power (Piantoni et al., 2021). Therefore, if female rats had greater vmHRV than male rats and vagal influences are also reflected in SDNN values and LF power, then it is not surprising that these HRV parameters were higher in female than male rats.

Our second hypothesis that the association between vmHRV and measures of cardiac chronotropy would be stronger in female than male rats was not fully supported by the current results. Specifically, female rats showed significantly stronger association between SDNN and both HR and IBI, replicating human findings (Williams et al., 2022). On the other hand, sex differences in the association between other HRV metrics (RMSSD, HF and LF) and indexes of cardiac chronotropy did not reach statistical significance, unlike in humans (Williams et al., 2022). Nevertheless, in comparing the current results in young adult rats with those reported in the sample of young adult humans (Williams et al., 2022), we ought to underline two important aspects. First, associations between vmHRV and indexes of cardiac chronotropy seem, in general, stronger in humans than rats. For example, the correlation coefficient for the association between RMSSD and HR was −.636 in humans (Williams et al., 2022) and −.327 in this sample of rats. However, in humans the analysis was conducted on a 5-min recording period during which participants sat in a resting position (Williams et al., 2022) and presumably vagal modulation had a stronger influence on cardiac chronotropy, whereas in rats the analysis was conducted on 24-h recordings during which rats were free to move and behave. Therefore, it would be interesting to investigate whether the associations between 24-h indexes of vmHRV and cardiac chronotropy in humans are weaker than those reported for 5-min baseline recordings and similar to those reported here in rats. The second aspect relates to sex differences in correlation coefficients. For example, the association between RMSSD and HR was significantly stronger in women (−.698) than man (−.560) in the previously studied large sample (n = 628) of young adult participants (Williams et al., 2022). In this smaller sample of young adult rats (n = 132), the magnitude of this sex difference was somewhat replicated (females = −.534 vs. males = −.394) and failure to reach full statistical significance may be due to insufficiently powered analysis.

### 4.1 Implications for translational research

Nowadays, the use of HRV as a polyvalent prognostic tool and reliable health indicator spans across researchers and practitioners from many different fields (Laborde et al., 2022). HRV is also assessed in rat models to increase the knowledge of the role of cardiac vagal modulation in several (patho)physiological processes (e.g., Sgoifo et al., 1997; Wood et al., 2012; Lee et al., 2013; Carnevali and Sgoifo, 2014; Chuang et al., 2017; Morais-Silva et al., 2019). To comprehensively appreciate the translational value of HRV measurement in rats, it is imperative that core biological characteristics that influence HRV in humans have the same effects in rats. Here, we demonstrate that biological sex influences the relationship between vmHRV and cardiac chronotropy in rats in a way that resembles the difference described between women and men. Therefore, the current results i) support the translational value of HRV findings in rat models, and ii) strongly recommend preclinical researchers to ensure that the HRV data obtained in rats of one sex are not generalized to both sexes, or, when possible, use both males and females. Further, studies in rats have shown that i) vagal nerve stimulation leads to greater cardiac effects in female than male rats, presumably due to a higher level of ACh release following nerve activation (Du et al., 1994), that ii) the enzymatic breakdown of ACh occurs slower in newborn female than male rats (Loy and Sheldon, 1987), and that iii) the synthesis and clearance of neurotransmitters, including ACh, in both the heart and vasculature are regulated by sex hormones (Dart et al., 2002). Intriguingly, a recent study (Leung et al., 2021) explored sex differences in the rat intrinsic cardiac nervous system, which includes the network of the intracardiac ganglia and interconnecting neurons that receive inputs from both local afferent and extrinsic autonomic (vagal and sympathetic) nerves (Achanta et al., 2020). The authors found that female rat hearts had fewer neurons and lower packing density than males, which may explain some of the sex differences observed at a functional level (Leung et al., 2021). Given such findings and the current results, we believe that rat models offer an important opportunity to systematically investigate the neuro-hormonal basis of the sex difference in the relationship between vmHRV and cardiac chronotropy. Importantly, an age-dependent decline in HRV has been described in humans and, similarly, cross-sectional evidence suggests that HRV is reduced in aged male rats and mice (Rossi et al., 2014; Piantoni et al., 2021). Therefore, given the relatively short lifespan of rodents compared to other animal species, it would be interesting to adopt longitudinal protocols in rodents to consider how sex differences in the association between HRV and cardiac chronotropy may be more or less evident with advancing age and the role of sex hormones.

### 4.2 Conclusion

Overall, the current study in a relatively large sample of freely moving young adult rats provides data illustrating a sex-dependent association between vmHRV and indexes of cardiac chronotropy. In particular, female rats showed greater vmHRV and higher HR than male rats, replicating the sex paradox described in humans (Koenig and Thayer, 2016), and a relatively stronger association between HRV and HR, as observed in young adult humans (Williams et al., 2022). We acknowledge that these results were obtained in wild-type Groningen rats and should be confirmed in different rat strains and potentially in other animal species, such as rabbits, with a more similar cardiovascular system to humans to increase their validity and generalizability. Moreover, the analysis was conducted on ECG signals recorded during undisturbed, baseline conditions to exclude the effects of environmental factors (e.g., stress exposure) on HRV measures, but the potential influence of trait behavioral characteristics was not considered. Nevertheless, the current results support the translational value of HRV findings in rat models and suggest researchers to always consider biological sex in the analysis and, most importantly, interpretation of HRV data in rats. Lastly, the present results represent, in our view, a solid starting point for a systematic investigation of the neuro-hormonal basis and temporal evolution of the impact of biological sex on the association between vmHRV and cardiac chronotropy in rats, which could inform the human condition.

## Data Availability

The original contributions presented in the study are included in the article/supplementary material, further inquiries can be directed to the corresponding author.
